# Effect of Pitch Angle on the Strength of a TC4/Helicoidal Composite Double-Bolt Scarf Joint: A Numerical Study

**DOI:** 10.3390/ma18173956

**Published:** 2025-08-24

**Authors:** Chunhua Wan, Xin Du, Guofan Zhang, Zhefeng Yu, Xin Lian

**Affiliations:** 1 National Key Laboratory of Strength and Structural Integrity, Aircraft Strength Research Institute of China, Xi’an 710065, China; wanc_1@163.com (C.W.); michaelzgf@163.com (G.Z.); 2 Aerospace Structure Research Center, School of Aeronautics and Astronautics, Shanghai Jiao Tong University, Shanghai 200240, China; xindu_sjtu@sjtu.edu.cn (X.D.); 412146266@sjtu.edu.cn (X.L.)

**Keywords:** scarf joint, helicoidal composite, progressive damage model, failure behavior

## Abstract

A progressive damage model was developed to study the damage and failure behavior of CFRP/Ti double-bolt scarf joints under quasi-static loading. The three-dimensional Hashin failure criterion was integrated into a finite element model via the ABAQUS user-defined material subroutine. Quasi-static tensile tests were conducted to investigate failure mechanisms and validate the model. The predicted failure modes match the experimental results with an error of 11.8% in the prediction of ultimate load. The effect of helicoidal layup on the composite joint was studied for the application of a helicoidal composite. The results show that the helicoidal layup configuration with a 45/−45 layup on the surface had the highest failure load, and the helicoidal layup introduced more tensile damage in the matrix. This study offers practical failure prediction methods and comprehensive failure mode analysis for composite bolted scarf joints.

## 1. Introduction

The application of mechanical multi-pin joints, including bolted joints and riveted joints, in composite materials is a commonly used connection method for engineering structures. This method is particularly prevalent when connecting critical load-bearing composite structures in aircraft. However, this leads to a more complex load distribution near the joints, thereby imposing higher demands on stress analysis and structural design in the connection area [1].

Owing to the high sensitivity of the ply angle in composite lap joints, this phenomenon will affect the effective stiffness and bearing capacity of riveted composite assemblies [2]. In a study by Franco [3], it was shown that angle ply ([0/90/(±45)_2_]_s_) laminates could nearly double the energy absorption of riveted joints compared to other configurations, and they had a higher ultimate load. Meanwhile, in the work of Seyednourani et al. [4], a laminate with a cross-ply configuration was found to possess a higher ultimate load than a laminate with an angle ply layup in sequence [45/−45]_4s_. Joint testing revealed that the failure modes of the pins included rivet shear, rivet tilt, and rivet pull-out [5]. Excessive interference in composite riveted joints can result in exceedance of the elastic deformation limit, leading to initial damage. To maintain the integrity of the joint, recommendations specify that the maximum interference size should not exceed 2% [6]. The effects of ply thickness and ply angle on the ultimate load of bolted joints have also been studied [7].

**Figure 1:**
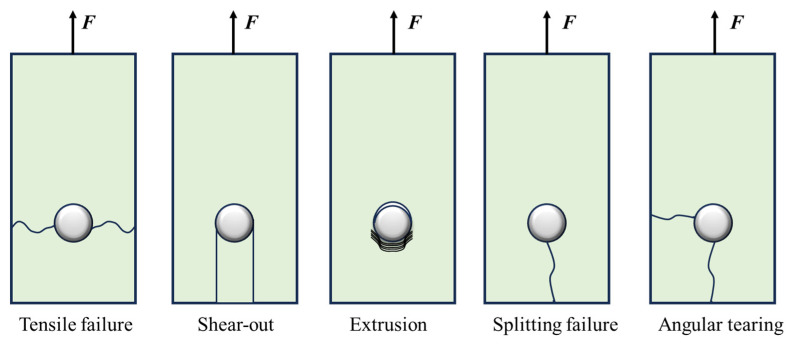
Failure mode of composite material in bolted joint.

The anisotropy and brittleness of composite materials, coupled with their laminated structures, result in significant stress concentrations in composite bolted joints. These factors lead to complex fracture behaviors and multiple failure modes. As shown in Figure 1, the primary failure mechanisms of composites in bolted structures can be categorized into tensile failure, shear failure, extrusion failure, and combined failure modes [8]. In addition, the delamination that occurs on the interface between layers is also considered for bolted joints. Given these failure mechanisms, the reinforcement design of composite bolted joints should focus on two key objectives: (1) increasing tensile strength, for example, by increasing the proportion of 0° fiber layers, and (2) ensuring that extrusion failure remains the dominant failure mode. An optimal reinforcement design should balance enhancing the joint strength while preserving extrusion as the preferred and safer mode of failure.

To address the aforementioned issues, it is essential to model the rivets that serve as the joint connectors. Rivets can be modeled using different methods [1], such as those using linear springs, nonlinear springs, and nonlinear beam elements. Some finite element programs (such as MSC.NASTRAN) provide bushing elements [9]. In a study by Pitta [10], rivets were modeled as point-based fasteners. The properties of the rivets were considered bushing elements with stiffness in six degrees of freedom: three in the translational direction and three in the rotational direction. The stiffness in the translational and rotational directions of bushing elements was calculated using closed-form solutions. Godzimirski [11] searched for the most effective ways of joining composites, including the use of different fasteners, such as aluminum rivets, Hi-lok, and Jo-Bolt, which differed not only in terms of the materials they were made of but also the pressure forces they exerted on the joined parts. Belardi et al. [12] evaluated the capabilities of joint elements and a solid model in simulating the multi-column in single-lap composite bolted joints, in which the former showed high efficiency.

In addition to constructing the rivet model, it is also essential to develop an efficient and accurate laminate model to replicate the failure process of composite material joints. From a meso-scale perspective, a composite laminate can be viewed as a three-dimensional structure consisting of multiple stacked layers. Therefore, Camanho and Matthews [13] proposed a three-dimensional finite element analysis in which the delamination onset criterion was applied at each interface. Djebbar et al. [14] investigated the effect of beveling a substrate’s geometrical shape on stresses in a bonded single-lap joint. The adhesive mesh consists of a single layer of eight-node three-dimensional cohesive elements, which was proven to be applicable to single-lap joint analysis. In a study by Wang [15], the general 3D Hashin [16] failure criterion and the stiffness degradation rule proposed by Tan [17] were employed to simulate the progressive failure behavior of a glass fiber/polypropylene riveted single-lap joint. Laminates and glass fiber/polypropylene rivets were simulated using linear, eight-node, 3D reduced integration elements. Cohesive elements were inserted into two parts of the model. The detailed intralaminar damage, including matrix cracks and fiber failures, can be predicted using the 3D Hashin criteria, which are generally applied to the material through the coded user-defined material subroutine (VUMAT) in ABAQUS/Explicit. The interlaminar damage (primarily delamination) is simulated using cohesive zone elements [18,19]. In the work of Cabrera-González et al. [20], several finite element analyses and experimental tests were carried out for basic load cases, different materials, and different orientations of the fiber. A tool was provided, thus allowing the users to easily estimate the stress distribution ($\sigma_{rr}$, $\sigma_{\theta \theta }$) along the hole contour and $\sigma_{xy}$ along the tangential lines to the hole circumference, which can be used for failure predictions. The nonlinear behavior of a single-lap bolted joint of woven glass fiber-reinforced plastic (GFRP) laminates was simulated in the work of Mohapatra et al. [21]. This nonlinear behavior was caused by shear failure and the damage of woven GFRP laminates. The results of the numerical simulation and experimentation matched well. Shan et al. [22] proposed a novel numerical prediction method based on a similar model to predict the fatigue failure behavior of a CFRP–aluminum two-bolt, double-lap joint.

In terms of joint modeling, this study focuses on the scarf joint of a helicoidal composite [23], which has lamination sequences that were inspired by naturally occurring impact-resistant Bouligand structures [24,25]. Bouligand-featured structures can be found in a variety of animals, including mantis shrimp [26], and they can also be found in fish scales [27] and the exoskeleton of arthropods, such as beetles [28]. The last segment of the appendage consists of several repetitions of the Bouligand unit stacked up through the thickness [29]. Each Bouligand unit contains a helicoidal layup with very small pitch angles for a rotation of 180° inside each unit. Research in the field of biomechanics has shown that cracks growing in the matrix of helicoidal composite specimens follow the fiber orientations and lead to the formation of twisted cracks [25]. A twisted crack growing in the helicoidal architecture amplifies the crack surface per unit volume, thereby enhancing energy dissipation and stress relaxation in the composite without leading to catastrophic failure. Some interlaminar cracks still exist; however, they are regarded as sub-critical delamination rather than the large delamination occurring in ordinary composites. In prior studies, the compressive strength after the impact of helicoidal composites mixed with additional 0° plies and the buckling optimization of curved grid-stiffened panels were investigated [30,31]. With their superior impact resistance, helicoidal composite materials may be applied in engineering structures in the future.

The strength of composite materials after repair is an important performance metric of concern, so the strength of spiral composite materials after repair needs to be studied. Scarf repair plays a significant role in composite laminate repair, and this study focuses on the prediction of the failure mode and failure load of metal/composite scarf joints with double countersunk head bolts. Due to its capabilities in nonlinear analysis, damage definition through the subroutine method, and interlaminar damage simulation, ABAQUS software version 6.14 was employed in the simulation. A progressive damage model, integrated with the enhanced 3D Hashin failure initiation criterion and stiffness degradation, was utilized to analyze the damage evolution in the composite laminate. A normal failure delamination model was developed in the out-of-face direction of the laminate. This approach deviates from the widely used zero-thickness cohesive zone model (CZM), which is typically applied in predicting the failure of carbon fiber-reinforced plastic (CFRP) interfaces and adhesive layer damage. This alternative model enables a more detailed analysis of the mechanical properties of composite laminates through the thickness. The intrinsic properties of the composites were incorporated via the development of a VUMAT subroutine. The progressive damage model was validated with experimental data, and it was utilized to investigate the influence of layup changes on the failure load, particularly in helicoidal laminates.

The remainder of this paper is structured as follows. In Section 2, the specimen and test setup are presented. The damage initiation criteria, damage evolution model, and the validation of the TC4/CFRP scarf joint are described in Section 3. In Section 4, a failure analysis of bolted joints and laminates is presented, and the effect of ply angle is also demonstrated using laminates with a helicoidal layup. Lastly, the key contributions are summarized and potential directions for future work are outlined in Section 5.

## 2. Configuration of the Joint Test Schedule

**Figure 2:**
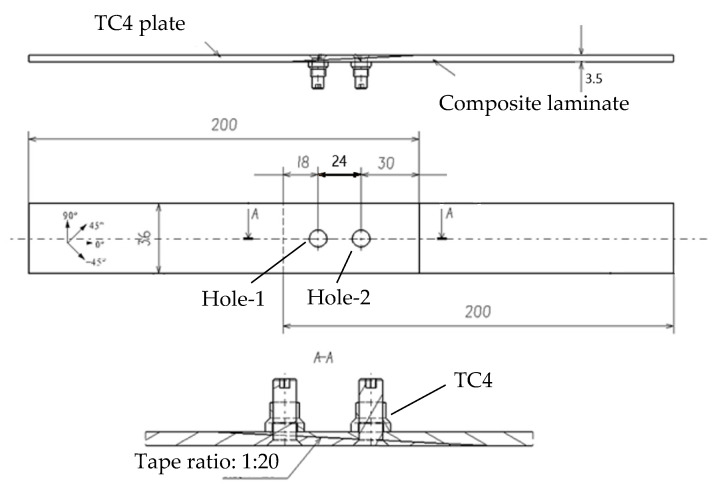
Configuration of the CFRP/TC4 joint.

To investigate the damage and failure modes of composite/metal double-bolted, single-shear scarf joints, specimens were designed in accordance with ASTM D5961 standards [32]. The specimens were fabricated using 5228A/CCF300 CFRP laminates and TC4, as shown in Figure 2.

The laminate is attached using single-shear, scarf joints. The CFRP laminate has a length of 200 mm and a width of 36 mm, with an edge-to-hole center distance of 18 mm and a spacing of 24 mm between the two holes. The total thickness of the laminate is 3.5 mm, with each layer being 0.125 mm thick. This laminate consists of four distinct ply orientations. Based on principal stress theory, 0° fiber layups are essential for bearing structural tension and compression. To minimize matrix loading in all directions, 90° layups are used sparingly. Additionally, ±45° layups are optimized for the outer surfaces to enhance structural stability and reduce coupling effects. Consequently, the layup sequence is formulated as [45/0/−45/−90/45/0_3_/−45/90/45/0_2_/−45]_s_.

The metal splice plates are made of a TC4 titanium alloy with a thickness of 3.5 mm. All bolts and gaskets within the joint structure are also constructed using the titanium alloy. The composite laminates and metal plates are joined with countersunk bolts. The shearing bolts, with countersunk heads angled at 100° and a diameter of 6 mm, were designed for precise tightening to ensure an ideal fit between the bolts and holes.

Uniaxial tensile tests were performed in compliance with the ASTM D5961 standard. To prevent slippage during loading, reinforcing plates were installed at both ends of the specimen. The specimen was firmly clamped in the grips of an electronic universal tensile testing machine. The bottom end of the bolted joint specimen was fixed, while a quasi-static displacement load was applied to the upper end. Throughout the testing process, load–displacement data were recorded in real time using a computer.

## 3. Numerical Simulations

### 3.1. Damage Initiation Criteria

The intralaminar damage was simulated using the ABAQUS/Explicit solver through a user material VUMAT subroutine developed in the FORTRAN language. Failure initiation was determined based on the Chang–Lessard failure criteria [33,34], which include four failure modes: matrix crushing, matrix cracking, fiber–matrix shearing failure, and fiber failure. In this study, fiber compressive failure was also considered. The Johnson–Cook damage model was employed to capture the damage behavior of the titanium alloy. The failure criteria are listed in Table 1. The strength parameters of the composite laminate and titanium alloy are listed in Table 2. The parameters of the former were determined by referring to similar materials employed in prior work [35] for parameter analysis, and those of the latter were adopted from a test conducted by a material research institute.

### 3.2. Model of Damage Evolution

**Figure 3:**
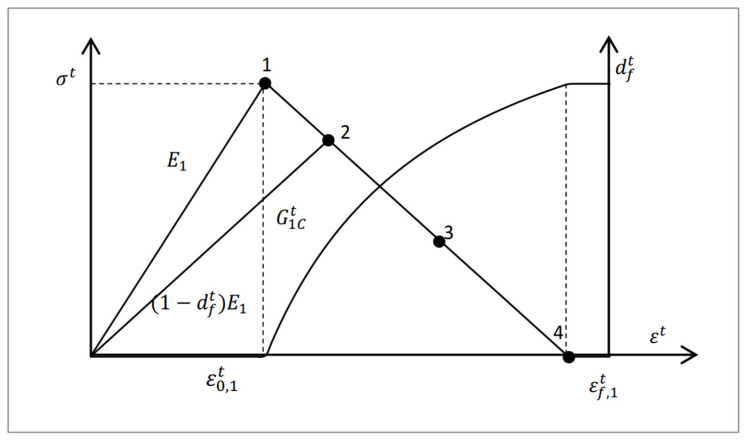
Intralaminar damage behavior model for tensile failure mode.

A linear stress–strain behavior was assumed for the composite laminar damage, as demonstrated with the tensile stress shown in Figure 3. Once the damage was initiated, the stress began to be linearly reduced to zero as the tensile strain increased. The failure initiation strain in tension $\varepsilon_{0,1}^{t}$ is expressed as follows:

(1) $$\varepsilon_{0,1}^{t}=\frac{X_{T}}{E_{1}}$$

The maximum strain $\varepsilon_{f,1}^{t}$ is given as follows:

(2) $$\varepsilon_{f,1}^{t}=\frac{2G_{1C}^{t}}{\sigma_{t}l}$$

where $G_{1C}^{t}$ is fracture toughness, which equals the area below the strain–stress curve, including the elastic and failure procedure; $\sigma^{t}$ is the ultimate tensile strength of the material; and l is the characteristic length for crack growth, which is relative to the element volume, as the fracture energy is distributed over the volume of the represented element. In the VUMAT subroutine, the cube root of the element volume was transferred through the variable *charLength*. The element had the same width and length, and the thickness t was 0.2 mm; thus, $l=\sqrt{{CharLength}^{3}/t}$.

The damage parameter for the respective failure mode is given by Faggiani et al. [36]. However, the damage variable in the tension of the fiber was modified, including the effect of shear strain, to improve a crash simulation of a composite tube as follows [35]:

(3) $$d_{f}^{t}=\frac{\varepsilon_{f,1}^{t}}{\varepsilon_{f,1}^{t}-\varepsilon_{0,1}^{t}}\left(1-\frac{1}{r_{ft}}\right)$$

where

(4) $$r_{\mathrm{ft}}=\sqrt{{\left(\frac{S_{11}}{\varepsilon_{0,1}^{t}E_{1}\left(1-d_{f,\mathrm{old}}^{t}\right)}\right)}^{2}+{\left(\frac{\varepsilon_{12}}{\varepsilon_{12}^{c}}\right)}^{2}+{\left(\frac{\varepsilon_{13}}{\varepsilon_{13}^{c}}\right)}^{2}}$$

where $d_{f,old}^{t}$ is the damage coefficient in the last step, and $\varepsilon_{12}^{c}\;and\;\varepsilon_{13}^{c}$ are the strains at damage initiation. The same damage evolution was applied to the damage of the unidirectional tape in two and three directions. The material stiffness matrix could then be obtained as follows [37]:

(5) $$C_{d}=\left[\begin{array}{cccccc} \alpha C_{11} & \alpha \beta C_{12} & \alpha \psi C_{13} & 0 & 0 & 0\\ \alpha \beta C_{12} & \beta C_{22} & \beta \psi C_{23} & 0 & 0 & 0\\ \alpha \psi C_{13} & \beta \psi C_{23} & \psi C_{33} & 0 & 0 & 0\\ 0 & 0 & 0 & \alpha \beta C_{44} & 0 & 0\\ 0 & 0 & 0 & 0 & \alpha \psi C_{55} & 0\\ 0 & 0 & 0 & 0 & 0 & \beta \psi C_{66} \end{array}\right]$$

where $\alpha =\left(1-d_{f}^{t}\right)\left(1-d_{f}^{c}\right)$; $\beta =\left(1-d_{m}^{t}\right)\left(1-d_{m}^{c}\right)$; $\psi =\left(1-d_{d}^{t}\right)\left(1-d_{d}^{c}\right)$; superscripts t and c denote tension and compression, respectively; and subscripts f, m, and d denote the one, two, and three directions of the unidirectional tape, respectively. The maximum of every damage variable was limited to approximately 0.8 to avoid the distortion of elements and maintain enough stiffness for simulating the supporting effect of composite debris. The element was deleted to avoid energy divergence when the strain was greater than 0.8 or less than −0.6, which are values that were determined through simulations and a comparison with the experimental results.

### 3.3. Finite Element Model

**Figure 4:**
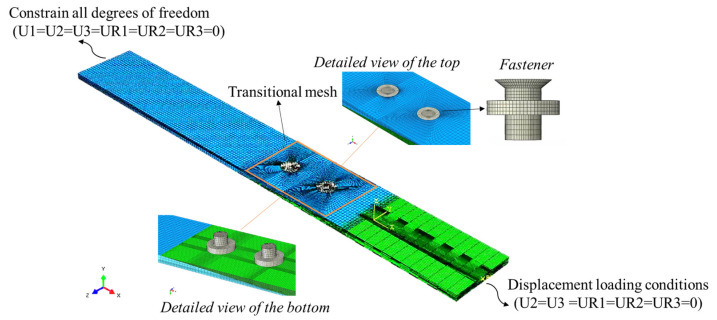
FE mesh and loading and boundary condition of bolted joints.

The finite element model, illustrated in Figure 4, was developed using ABAQUS, with the material properties detailed in Table 2.

The accuracy of numerical simulations is highly dependent on mesh quality, thus making precise meshing essential in critical regions. The experimental results reveal that stress concentrations are the most severe around the hole circumference, with failures rarely occurring in regions farther from the hole edge. To address this, a refined mesh density was applied near the hole, with a gradual transition to coarser meshes in less critical areas. There are 38 elements around the whole periphery of the fastener hole, which is similar to the mesh convergence results of Rosales-Iriarte et al. [38] and Liu et al. [39]. This meshing strategy, validated through the simulation results, ensures both reliable accuracy and acceptable computational time, with each simulation taking about 18 h on a workstation equipped with an Intel Core i7-13700F processor (Santa Clara, CA, USA).

Each layer of the composite material was modeled as a single element (C3D8R) along the thickness direction to accurately capture the stress state and damage behavior of the laminate at the individual layer level. The damage evolution criterion of the laminate, as outlined in Section 3.2, was implemented using the VUMAT subroutine.

Countersunk bolts were modeled in a simplified manner, excluding the effects of threading. The interaction between the two plates and the bolt was defined as face-to-face contact with finite slip. Contact behavior was specified as “hard contact,” and the overall friction coefficient was set to 0.2. Boundary conditions restricted transverse displacement (U2) and out-of-plane displacement (U3), fixed the left end, and applied axial displacement at the right end (U1). Additionally, the amplitude of a smooth step function was employed to support the loading process.

The quasi-static loading process was simulated and solved using ABAQUS/Explicit, ensuring that the ratio of kinetic energy to internal energy related to kinetic energy and strain energy remained below 5% and maintaining stability and accuracy throughout the simulation.

### 3.4. Model Validation

**Figure 5:**
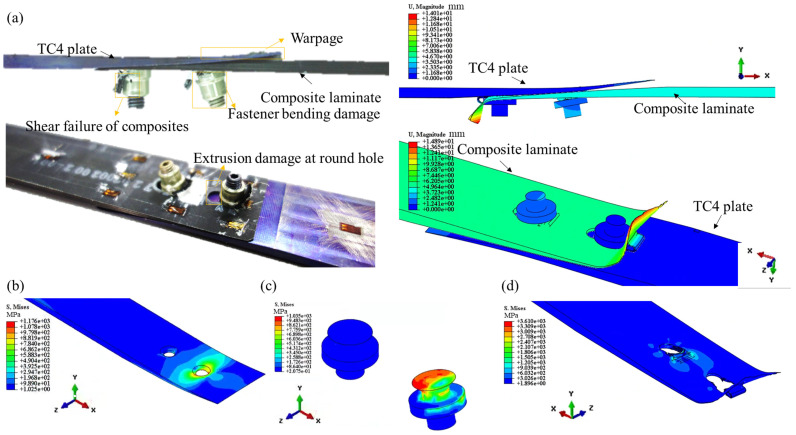
Damage modes of scarf joint specimen: (**a**) comparison of damaged specimen, (**b**) stress concentration at edge of TC4 plate hole, (**c**) bending of fastener, and (**d**) extrusion and shear failure in composite laminate.

The failure mechanism observed in the test is compared with the numerical simulation, as shown in Figure 5. The primary failure modes include extrusion damage around the bolt holes in the thin-walled composite laminate, the bending and cracking of the fastener head in the thin-walled metal repair plate, and the warping deformation of the thin metal repair plate. The corresponding simulation results are presented in Figure 5b–d, which show that the model is effective in predicting the progression of failure in the composite countersunk head bolted structure. This model serves as a reliable tool for studying structural damage and failure, thereby providing valuable insights into the failure mechanisms of composite bolted joints under mechanical loading conditions.

**Figure 6:**
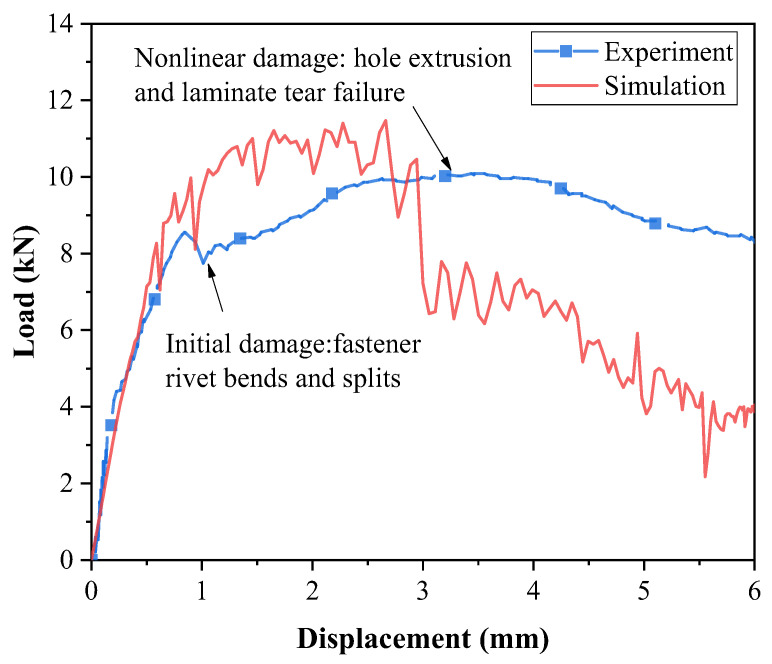
Typical load–displacement curves for single-shear diagonal lap static tests.

The typical load–displacement curves are illustrated in Figure 6. During the initial loading phase, the load–displacement curve exhibits a linear elastic response. In the absence of damage, the simulation results closely align with the experimental curve, thus demonstrating strong correlation. As displacement increases, the fastener experiences bending failure, causing the load curve to decline around 8 kN; however, the structure retains some residual load-carrying capacity.

With further displacement, the load rises to a peak value of 10.06 kN, at which point varying degrees of extrusion damage are observed at the hole edges of the composite laminate, with more pronounced damage localized near the holes on the thinner side. Eventually, the structure loses its ultimate load-bearing capacity entirely. Unlike the commonly observed bolt shear failure mode, the failure of titanium alloy fasteners is primarily characterized by laminate extrusion failure and the tension-induced bending of the fasteners.

The initial damage load of the simulation agrees with the experimental results. The simulated load increased to 11.40 kN in the following procedure, then dropped dramatically as shear off occurred. The maximum load of the experiment is 10.06 kN; thus, the error in the ultimate load prediction is 11.8%. However, obvious deviations occur in the extrusion phase compared to the experimental curve, which can be attributed to the challenges in numerical simulation based on the intrinsic complexities of this type of damage of composite laminates. For example, accurately determining material fracture energy involves significant costs, intricate procedures, and a high degree of uncertainty. Additionally, the extrusion failure process in composite bolted joints is highly complex and primarily driven by fiber compression and matrix failure. These factors contribute to the discrepancies observed in the load–displacement curves between the simulation and experimental results.

## 4. Failure Analysis and Discussion

### 4.1. Failure Analysis of Bolted Joints

#### 4.1.1. Local Deformation of Scarf Joints

**Figure 7:**
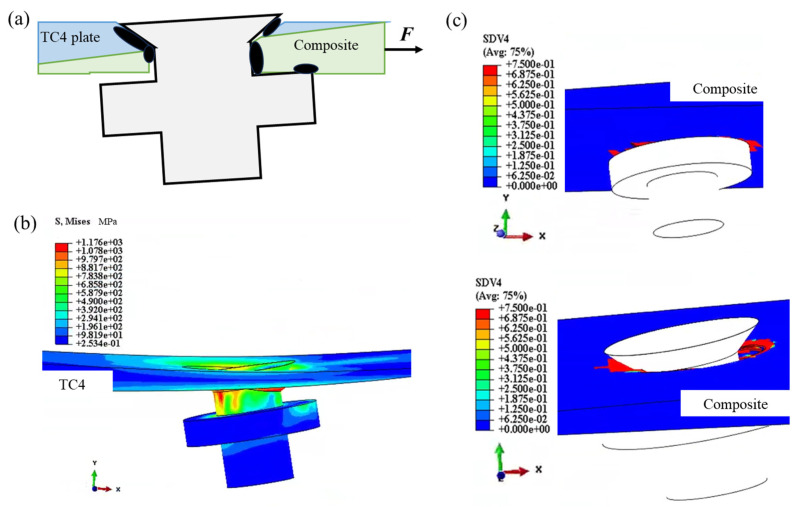
Local deformation and contact in countersunk bolted joints: (**a**) stressed areas, (**b**) stress in TC4 joint, and (**c**) matrix damage due to compression in composite joint.

Figure 7a depicts the local deformation and contact area between the fastener holes and bolts in the double-bolt scarf joint.

The introduction of countersunk holes leads to a local reduction in stiffness around the fastener holes in the composite plate. This stiffness disparity is further amplified by the thickness difference caused by the scarf angle, in which the bearing deformation at the upper plate holes is significantly larger than that at the lower plate holes. As a result, the countersunk bolts experience rotation, thus leading to the asymmetric bending of the bolt shanks and radial deformation along the through-thickness path.

As the load increases, the contact area between the upper plate and the bolt shank progressively shifts to the cylindrical section of the hole. This transition enhances the contact area on the upper plate, thus enabling it to more effectively resist the bearing load. Consequently, the localized deformation near the countersunk holes is reduced. Over time, this process mitigates the asymmetry in the bending of the countersunk bolts, thereby delaying structural failure until the ultimate load capacity is exceeded.

**Figure 8:**
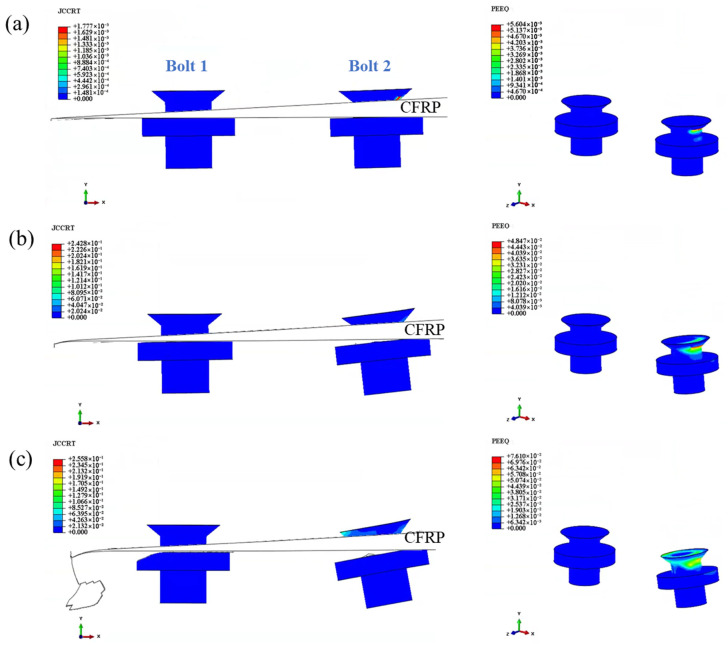
Bending damage and plastic deformation of bolts: (**a**) linear elasticity phase, (**b**) bending failure of bolt, and (**c**) final failure of test specimen, where JCCRT refers to Johnson–Cook damage criterion, and PEEQ refers to equivalent plastic strain.

As shown in Figure 8, the loading process induces both damage and plastic deformation in the countersunk bolts.

For bolt 1, in which the laminate is thinner, the contact area is mainly confined to the cylindrical holes. This leads to laminate extrusion and shear damage concentrated along the loading direction. In contrast, at bolt 2, the thicker laminate with greater load-bearing capacity primarily exhibits laminate extrusion damage, accompanied by bolt bending failure.

Throughout the loading process, bolt 2 plays a more significant role in the overall structural response due to its greater load-bearing capacity. This highlights the critical influence of laminate thickness and bolt interaction on structural performance and failure mechanisms.

#### 4.1.2. Stress Distribution Around Fastener Holes

**Figure 9:**
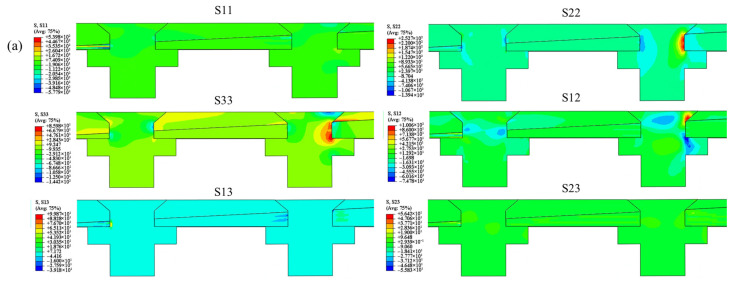

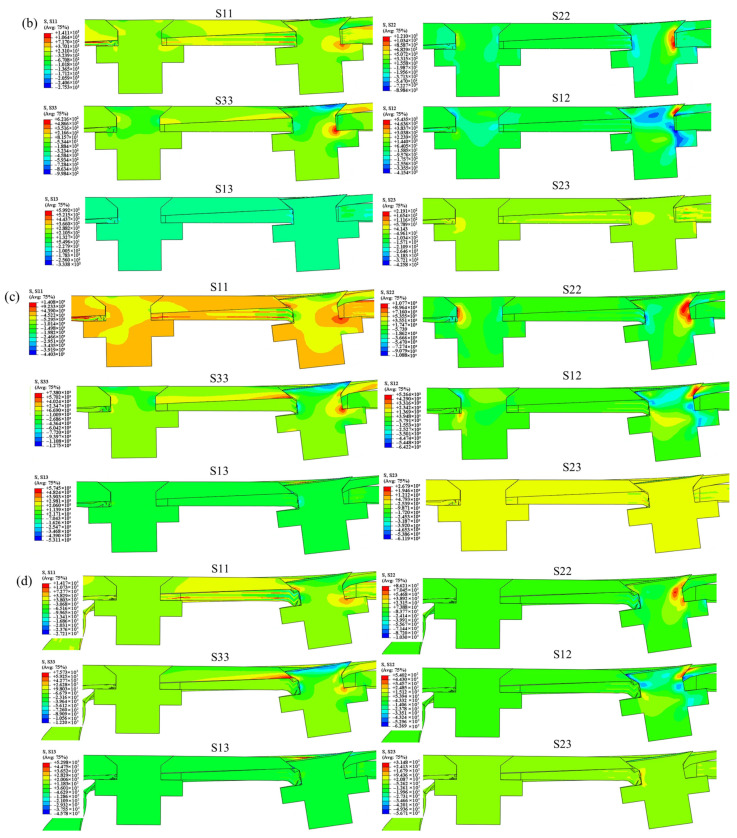
Stresses in countersunk joint: (**a**) linear elasticity phase, (**b**) bending failure of bolt, (**c**) at point of peak load, and (**d**) final failure of test specimen.

Typical stress contours around fastener holes in the double-bolt joints, as shown in Figure 9, reveal that critical stress zones for all components are concentrated either on the upper or lower side of the midplane.

In countersunk hole joints, stress components such as *S*_11_, *S*_22_, *S*_12_, and *S*_33_ are similarly distributed on the upper or lower side of the center plate. A key observation is that critical stress regions *S*_33_ and *S*_13_ are particularly located at the start of the countersunk hole in the upper plate, thus illustrating that countersunk holes introduce significant stress concentration in this region. This stress concentration may induce warpage in the upper plate (TC4).

For scarf joints with countersunk head bolts, stress concentrations are primarily found at the countersunk head end and near the threads. In contrast, the tapered portion near the nail experiences only minimal stress concentration. This creates a pronounced stress gradient between the screw and the countersunk head, making the top of the screw prone to significant deformation.

Additionally, the extrusion effect generated by the beveled surface of the countersunk head section may cause delamination damage in the composite plate. This delamination can intensify the sub-buckling phenomenon observed in the scarf joint structure, thus further affecting the joint performance and load-bearing capacity.

### 4.2. Failure Analysis of Plates

Based on the output variables, the progression of different damage modes in the composite plate can be systematically monitored. Initially, matrix compression and fiber compression damage are the primary damage modes, typically initiating at the 90° layer of the composite lap plate (hole 1), which is the closest to the lap surface. As loading increases, this damage gradually propagates to the inner layers of the lap plate. Subsequently, matrix tensile damage and fiber tensile damage also become evident.

**Figure 10:**
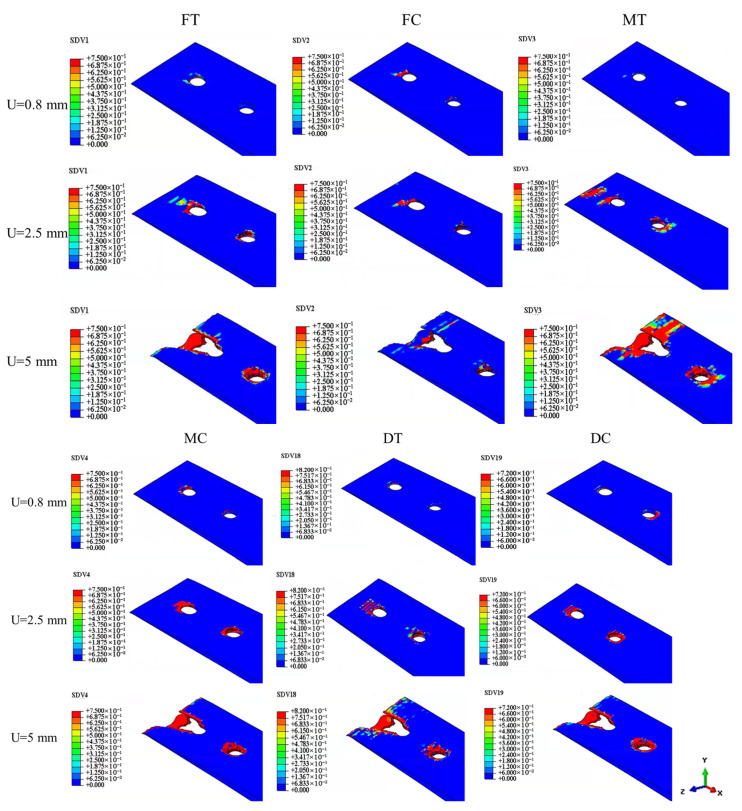
The progressive failure process of the laminate in the joint.

Once the damage progression accelerates, the damage variables in the composite laminate quickly reach their maximum values, and damage extends outward toward the periphery. Simultaneously, the state variable JCCRT (Johnson–Cook damage initiation criterion) of the bolt begins to show positive values, which steadily increase during loading. At the ultimate load stage, the overall damage states, as illustrated in Figure 10, identify key damage areas, including extrusion and shear-out damage at hole 1 and extrusion damage at hole 2. Furthermore, due to the deflection of bolt 2 during loading, Z-direction damage appears at hole 2.

A comparison of the simulated damage distributions with experimental damage photographs demonstrates strong agreement between the simulation results and test observations. This confirms the accuracy of the model in capturing the primary damage mechanisms, including extrusion, shear-out, and Z-direction damage, along with their propagation during the loading process.

**Figure 11:**
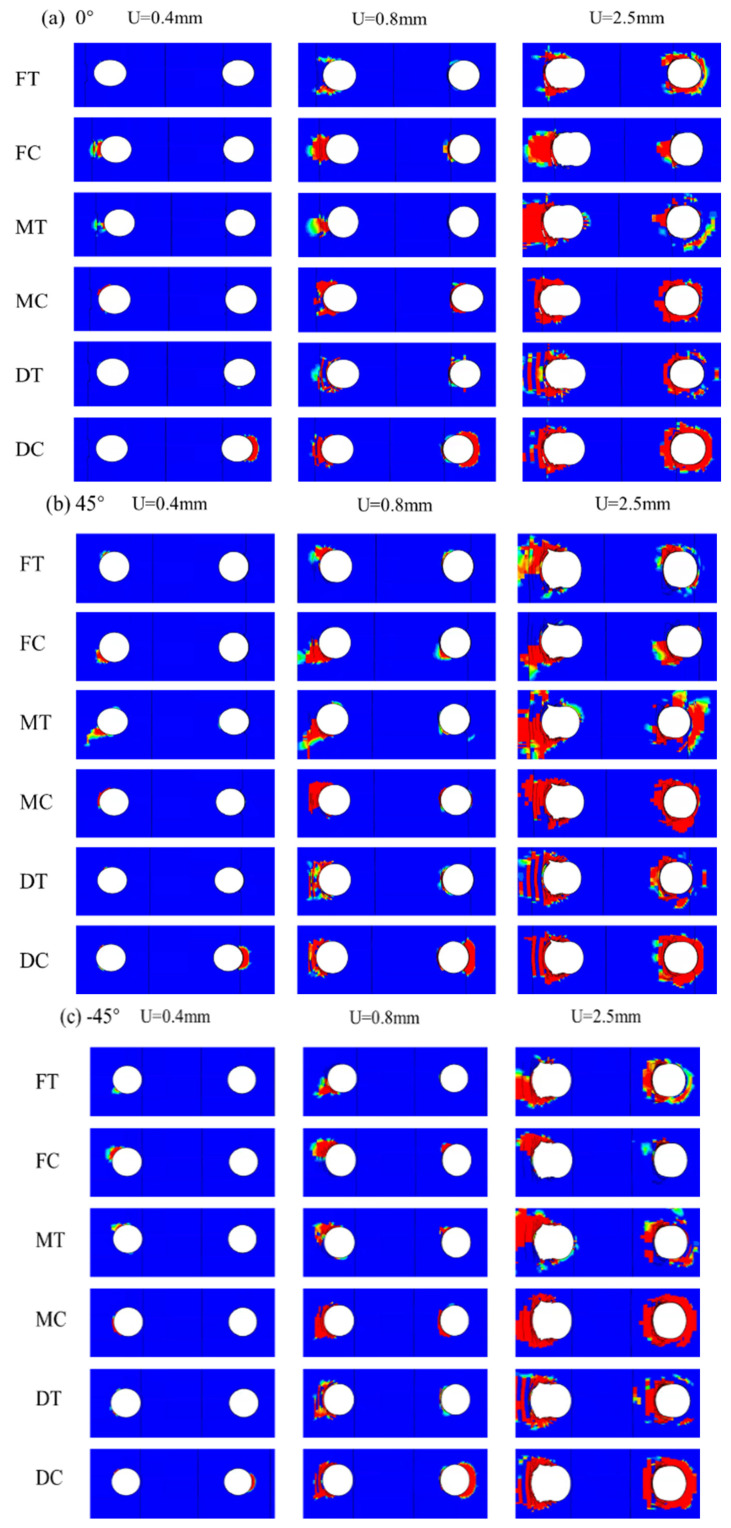

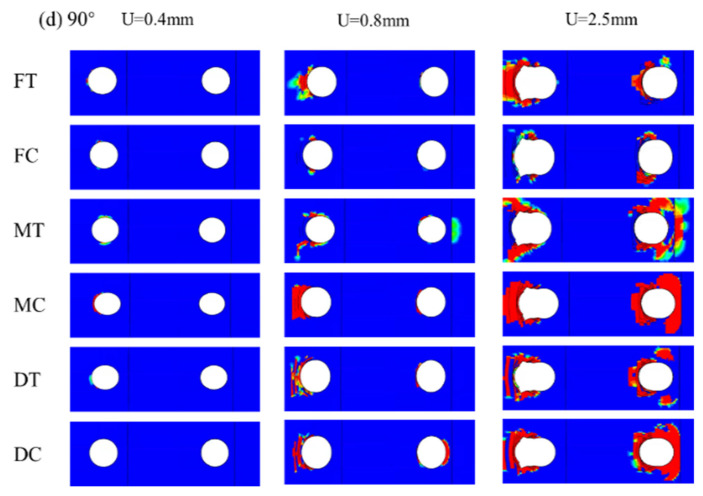
Progressive failure process of each layup angle laminate around hole: (**a**) 0° ply, (**b**) 45° ply, (**c**) −45° ply, and (**d**) 90° ply.

To understand the failure mechanism and guide structural optimization, analyzing the failure mode of individual layers within the connection structure is essential. Since tensile forces in the 0° direction dominate the radial connection, the 0° ply of the composite laminate plays a pivotal role in load-bearing. Figure 11 displays the failure modes for each laminate ply angle, with the progression of damage indicated by the damage variable *d*.

Areas shaded in red, representing the largest values of *d*, correspond to zones of the most severe damage, which align with stress concentrations near the hole perimeter.

At a displacement of 0.4 mm, fiber compression and matrix tensile damage begin to develop in localized regions around bolt 1. Initial tensile damage appears in the ±45° plies, while the 90° plies exhibit the lowest load-bearing capacity. This discrepancy reflects different load transmission mechanisms in various ply orientations. Specifically, the 0° plies are primarily loaded via the fibers, while the ±45° plies transmit loads through both the fibers and the matrix. Because the matrix has a considerably lower load-bearing capacity than the fibers, it is the first to fail. As displacement increases, fiber tensile damage emerges in the 0° ply, leading to the immediate loss of load-bearing capacity in the affected element and intensifying local stress concentrations. Since the fibers bear most of the tensile load, their failure significantly undermines structural reliability.

At a displacement of 0.8 mm, laminate delamination occurs due to bolt failure caused by bending. The bolts apply compressive forces to the screw holes, resulting in localized compressive collapse near the bolt holes. However, tensile damage remains the primary failure mechanism in the laminate. Matrix tensile failure precedes fiber tensile failure, with the damage propagating sequentially from the ±45° plies to the 90° plies, while fiber tensile damage spreads from the 0° plies to the ±45° plies. Since the fibers offer significantly higher load-bearing capacity than the matrix, their failure results in an immediate and substantial reduction in load-carrying capacity, thus critically impacting structural reliability.

As the displacement increases further, the damage zone continues to grow toward the laminate edges. This progressive expansion of the damage ultimately leads to a complete loss of structural load-bearing capacity, thus signifying total failure. By observing these failure patterns, it becomes evident that preventing fiber tensile failure is crucial to maintaining structural integrity and optimizing the connection design.

### 4.3. Effects of Helicoidal Layup

The potential application of helicoidal composites in engineering structures underscores the critical need to investigate and optimize their joint strength. By appropriately adjusting the orientation of the fiber layup, the superior mechanical properties of the fibers can be leveraged more effectively, thus enhancing the overall load-bearing capacity of the structure.

This subsection introduces an exploratory study focused on optimizing helicoidal layup angles for improved performance. The above specimen plays a role of a baseline (BL) in evaluating the impact of fiber layup orientations on the mechanical behavior and joint strength of helicoidal composites, as listed in Table 3. The laminate thickness for each specimen is 3.5 mm, and each specimen is composed of 28 layers, which are 0.125 mm thick. The composite material layering scheme was selected to ensure that the number of layers in each test piece is equal and the layering is symmetrical. For helicoidal composite laminates, a 180° layup is maintained in the center. An exception is made for specimen He16, which features a 45/−45 layup on the surface of the helicoidal layer to examine the protective effect of this conventional layer. The simulation results indicate that this configuration exhibits a relatively high ultimate load.

**Figure 12:**
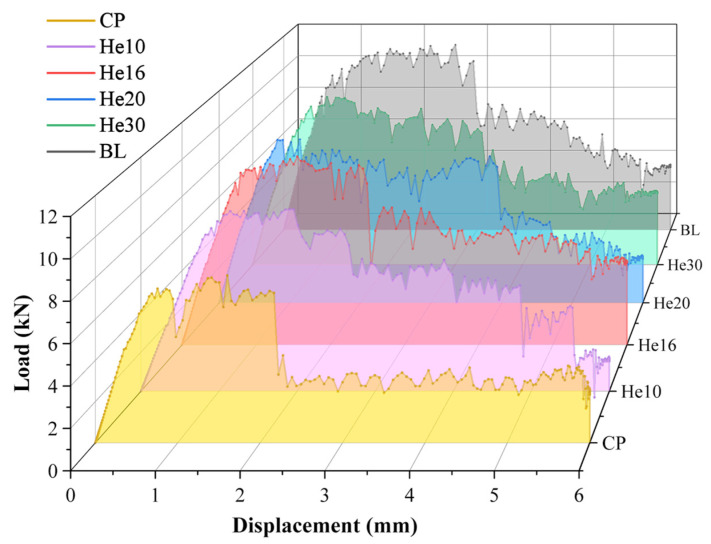
Load–displacement curves of different types of specimens.

The load curves for specimens of different layup configurations, which are presented in Figure 12, are compared to those of the benchmark test specimens discussed previously.

**Figure 13:**
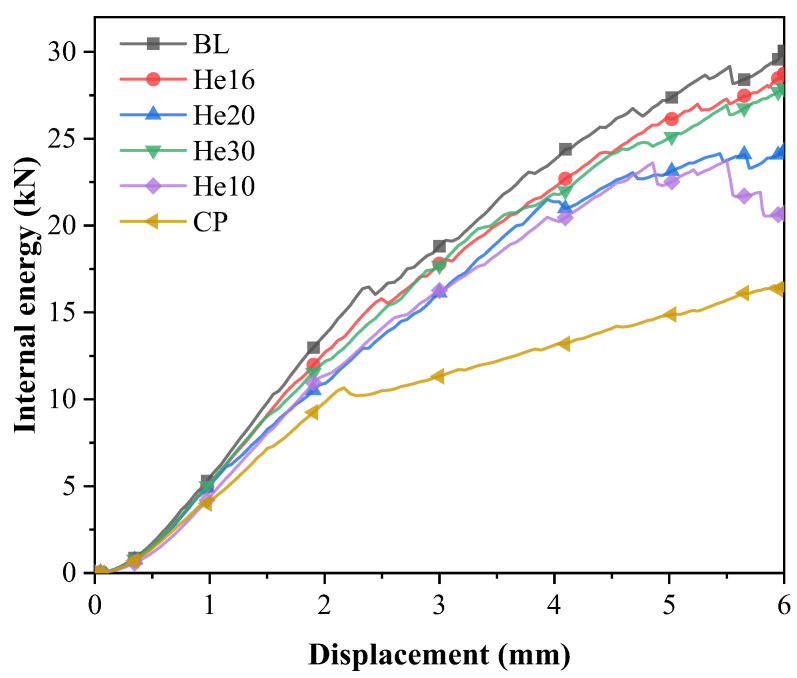
Internal energy–displacement curves of different specimens.

These load–displacement curves display a notable progression: an initial linear increase in load, which is followed by a load decline as the bending failure of the bolt occurs. The load then continues to increase but at a reduced rate. Ultimately, a series of load drops leads to structural failure. Insights into the tensile damage process within the scarf joint model are further provided by the internal energy variations during the simulation, as shown in Figure 13.

**Figure 14:**
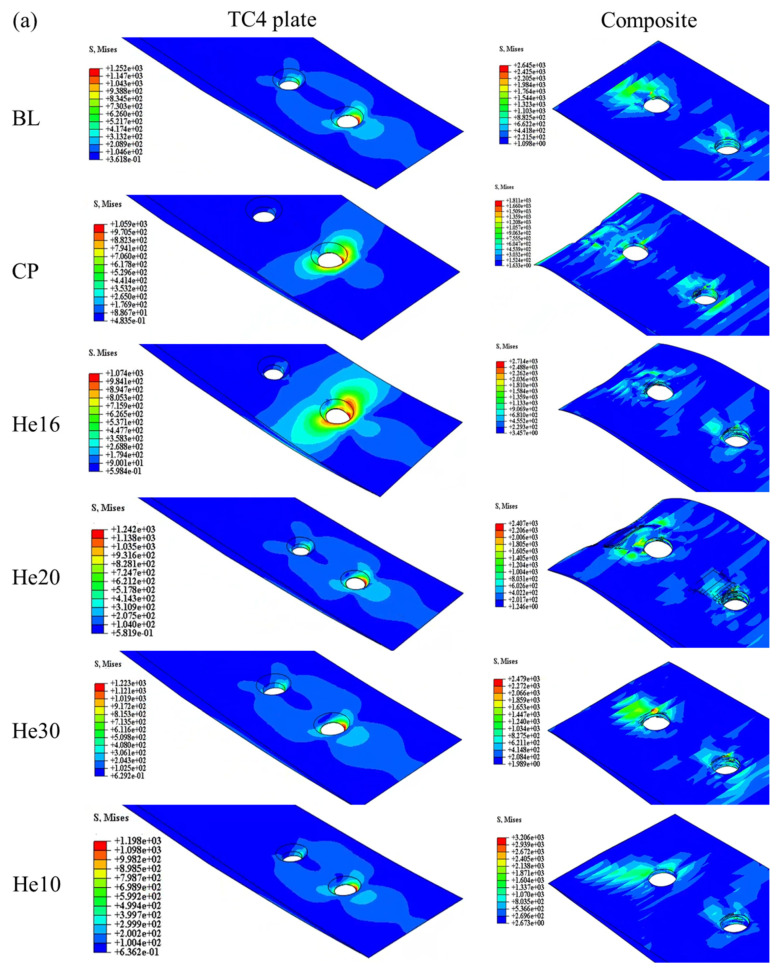

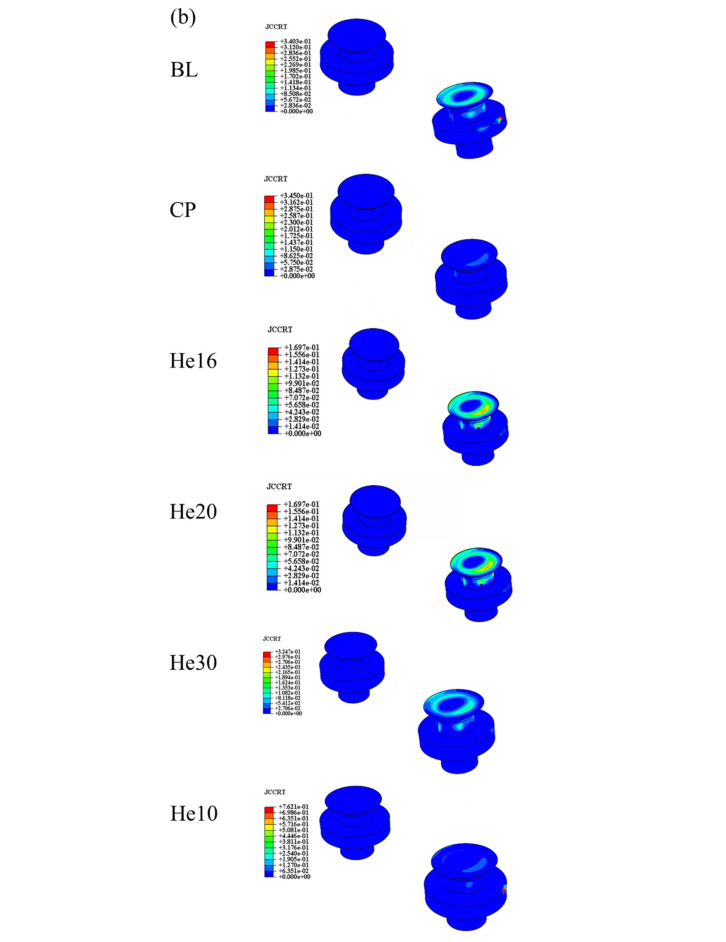
Damage of different specimens: (**a**) stress distribution of plate (MPa) and (**b**) bolt damage.

As shown in Figure 14, the stress distribution in the lap model at peak load reveals key areas of stress concentration. In the metal plate, stress concentrations are primarily localized around the bolt holes, whereas in the composite plate, stress concentrations occur both around the holes and in the wedge transition region. These regions are subjected to the highest mechanical demands during loading, thus making them critical to the structural integrity of the joint.

For the bolts, the stress concentrations are primarily associated with bending and extrusion damage, as illustrated in Figure 14b. The form of bolt damage in the He16 specimen closely resembles that of the BL specimen, indicating a similar load transfer and damage mechanism. Conversely, the He10 and CP specimens experience significantly less bolt damage due to the lower tensile loads borne by these configurations. This reduced tensile load prevents substantial stress accumulation in the bolts, thus limiting the extent of bending and extrusion damage in these specimens.

Overall, the He16 specimen stands out since it achieved a damage pattern comparable to that of the BL specimen, while the weaker tensile response of the He10 and CP configurations underscores their limited structural performance and reduced susceptibility to bolt damage. These insights highlight the critical role of joint configuration in distributing stress and influencing damage mechanisms within the scarf joint system.

**Figure 15:**
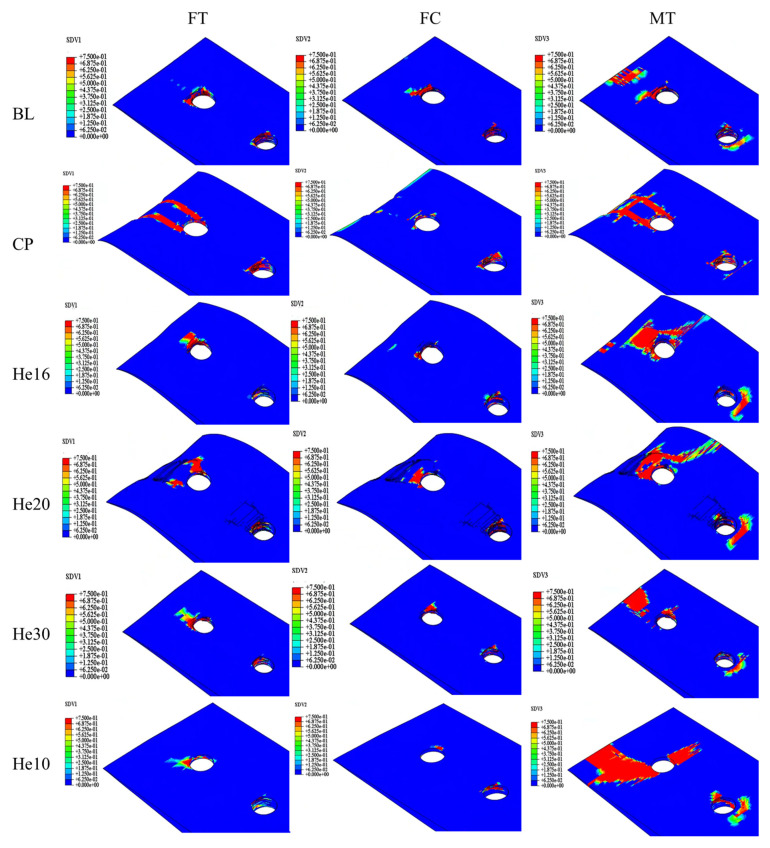

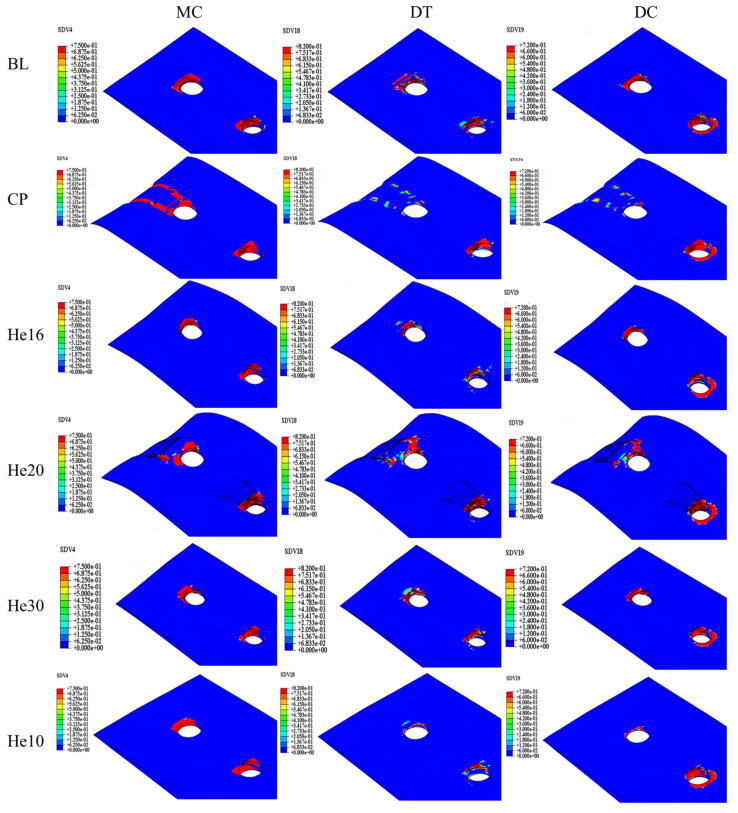
Damage distribution of different specimens under peak load.

The failure modes of each specimen are shown in Figure 15. The damage of shear-out is shown in hole 1 of the CP specimen, which exhibits the lowest peak load. The larger matrix tensile damage is shown in the helicoidal composite specimen. The peak load, displacement at peak load, and energy absorption at 6 mm are listed in Table 4.

The orthogonal layup CP configuration exhibits the lowest peak load, which is primarily due to the increase in transverse crack formation between the fibers oriented at a 90° angle during tensile loading. This susceptibility significantly reduces the load-carrying capacity of the structure. In the work of Seyednourani et al. [4], the laminate with a cross-ply possess a higher ultimate load than the one with an angle ply layup with the sequence [45/−45]_4s_ because shear failure between the plies of the latter easily occurs. The stress of composite bolted joints is very complex, and only when each layer can bear complex loads in multiple directions can a higher bearing capacity be achieved. As shown in the simulation results, the baseline model has the best performance. The BL specimen, however, achieves the highest peak load, the greatest elastic stiffness, and the most efficient energy absorption. This superior performance is directly linked to its fiber composition, which features approximately 42.9% fiber alignment in the 0° direction. This alignment optimally reinforces the structure and maximizes load-bearing efficiency, thus making the BL specimen the standout configuration in terms of mechanical performance.

Among the helicoidal configurations, He16 demonstrates a load-bearing capacity similar to that of the baseline specimen. This performance is attributed to its uniform 16° helix angle throughout a symmetric 28-layer design, supplemented by the 45°/-45° layup in the outermost layers, which enhances structural stability. Specimen He30 displays suboptimal mechanical properties, as its layup configuration is similar to that of the BL specimen. The peak loads of He10 and He20 are slightly lower. Helicoidal specimens perform better than CP specimens. These helicoidal configurations may mitigate substrate delamination, thereby preserving structural integrity by requiring more force to induce transverse cracking, which subsequently results in a higher peak force.

From these observations, it can be concluded that optimizing the interlayer angle has the potential to improve the overall structural performance, thus reducing fiber fracture to a secondary damage mode. Furthermore, no substantial differences in delamination damage were observed across the various ply types. The delamination observed was predominantly caused by bolt bending and the extrusion of screw holes, leading to localized failure.

## 5. Conclusions

In this research, we effectively combined the finite element method with a progressive damage model to evaluate the reinforcement design’s impact on composite bolted joint damage resistance. We have highlighted the reinforcement’s role in damage propagation and failure modes, providing a foundation for the experimental design and performance prediction of composite bolted joints. Using the 3D HASHIN criterion, a finite element model for the progressive damage analysis of a double-lap bolted joint was established. By comparing the simulation results with experimental data, the following conclusions were drawn:

- The model effectively simulated the load–displacement curve and accurately predicted the failure modes of the baseline specimen, with an error of 11.8% in the prediction of ultimate load.
- The local deformation of the composite layers around the fastener holes, combined with bolt bending, resulted in varied stress distributions in the joints.
- The effect of helicoidal layup on the composite joint was studied. The results show that the He16 configuration with a 45/−45 layup had the highest failure load. Helicoidal layup introduces more tensile damage in the matrix.

However, due to challenges in numerical simulation and the intrinsic complexity of the bolted joint model, discrepancies were observed between the failure-stage curves derived from finite element simulations and those from the experimental results. These differences highlight certain limitations in accurately modeling the intricate interactions within bolted joints during failure progression.

## Figures and Tables

**Table 1:** Failure criteria for the unidirectional ply.

Failure Modes	Damage Factors
Laminate	
$\mathrm{Fiber}\;\mathrm{tensile}\;(\mathrm{FT})\;\mathrm{failure}\;(\sigma_{1}\geq 0$)	$F_{ft}=\sqrt{{\left(\frac{\sigma_{1}}{X_{T}}\right)}^{2}+{\left(\frac{\tau_{12}}{S_{12}}\right)}^{2}+{\left(\frac{\tau_{13}}{S_{13}}\right)}^{2}}\geq 1$
$\mathrm{Fiber}\;\mathrm{compressive}\;(\mathrm{FC})\;\mathrm{failure}\;(\sigma_{1}<0$)	$F_{fc}=\sqrt{{\left(\frac{\sigma_{1}}{X_{C}}\right)}^{2}}\geq 1$
$\mathrm{Matrix}\;\mathrm{tensile}\;(\mathrm{MT})\;\mathrm{failure}\;\mathrm{in}\;2\;\mathrm{directions}\;(\sigma_{2}\geq 0$)	$F_{\mathrm{mt}}=\sqrt{{\left(\frac{\sigma_{2}}{Y_{T}}\right)}^{2}+{\left(\frac{\tau_{12}}{S_{12}}\right)}^{2}+{\left(\frac{\tau_{23}}{S_{23}}\right)}^{2}}\geq 1$
$\mathrm{Matrix}\;\mathrm{crushing}\;(\mathrm{MC})\;\mathrm{failure}\;\mathrm{criterion}\;\mathrm{for}\;\text{in-plane}\;\mathrm{compression}\;(\sigma_{2}<0$)	$F_{\mathrm{mc}}=\sqrt{{\left(\frac{\sigma_{2}}{Y_{C}}\right)}^{2}+{\left(\frac{\tau_{12}}{S_{12}}\right)}^{2}+{\left(\frac{\tau_{23}}{S_{23}}\right)}^{2}}\geq 1$
$\mathrm{Delamination}\;(\mathrm{DT})\;\mathrm{in}\;\mathrm{tension}\;(\sigma_{3}\geq 0$)	$F_{\mathrm{dt}}=\sqrt{{\left(\frac{\sigma_{3}}{Z_{T}}\right)}^{2}+{\left(\frac{\tau_{13}}{S_{13}}\right)}^{2}+{\left(\frac{\tau_{23}}{S_{23}}\right)}^{2}}\geq 1$
$\mathrm{Delamination}\;\mathrm{in}\;\mathrm{compression}\;(\mathrm{DC})\;(\sigma_{3}<0$)	$F_{\mathrm{dc}}=\sqrt{{\left(\frac{\sigma_{3}}{Z_{C}}\right)}^{2}+{\left(\frac{\tau_{13}}{S_{13}}\right)}^{2}+{\left(\frac{\tau_{23}}{S_{23}}\right)}^{2}}\geq 1$
TC4	
Equivalent flow stress σ	$\sigma =\left(A+B\varepsilon^{n}\right)\left(1+C\ln{\dot{\varepsilon }}^{*}\right)\left(1-T^{*m}\right)$ ${\dot{\varepsilon }}^{*}=\frac{\dot{\varepsilon }}{\dot{\varepsilon_{0}}}$ $T^{*}=\frac{T-T_{0}}{T_{m}-T_{0}}$
$\mathrm{Failure}\;\mathrm{plastic}\;\mathrm{strain}\;\varepsilon_{f}$	$\varepsilon_{f}=\left[D_{1}+D_{2}\exp\left(D_{3}\eta \right)\right]\left[1+D_{4}\ln\left(\frac{\dot{\varepsilon }}{{\dot{\varepsilon }}_{0}}\right)\right]\left[1+D_{5}\left(\frac{T-T_{0}}{T_{m}-T_{0}}\right)\right]$
$\sigma_{1},\sigma_{2},\;\sigma_{3}-$$\mathrm{normal}\;\mathrm{stress};\;\tau_{12},\tau_{13},\;\tau_{23}-$$\mathrm{shearing}\;\mathrm{stress};\;F_{k}$(*k* = damage factor: ft, fc, mt, mc, dt, dc).	

**Table 2:** Mechanical properties of carbon fiber-reinforced composite laminate and TC4.

Type	Parameter	Unit	Value
Laminate	Density	g/cm^3^	1.529
	*E*_1_	MPa	11,000
	*E*_2_	MPa	8000
	*E*_3_	MPa	8000
	$\nu_{12}$	-	0.3
	*G*_12_	MPa	4900
	*G*_13_	MPa	4900
	*G*_23_	MPa	3000
	$G_{1C}^{t}$	N/mm	133
	$G_{1C}^{c}$	N/mm	40
	$G_{2C}^{t}$	N/mm	0.6
	$G_{2C}^{c}$	N/mm	2.1
	$G_{3C}^{t}$	N/mm	0.6
	$G_{3C}^{c}$	N/mm	2.1
	*X*_t_	MPa	2688
	*X*_c_	MPa	1458
	*Y*_t_	MPa	69.5
	*Y*_c_	MPa	236
	*Z*_t_	MPa	55.5
	*Z*_c_	MPa	175
	*S*_12_	MPa	136
	*S*_13_	MPa	136
	*S*_23_	MPa	95.6
Titanium alloy	*E*	MPa	110,000
	*v*	-	0.31
	Yield stress *A*	MPa	955
	Strain hardening modulus *B*	MPa	250
	Hardening exponent of materials *n*	-	0.37
	Temperature effect m	-	1.1
	Melting temperature *T*_melt_	°C	1951
	Reference temperature *T*_ref_	°C	298
	*D*_1_	-	0.0835
	*D*_2_	-	0.657
	*D*_3_	-	−2.09
	*D*_4_	-	0.014
	*D*_5_	-	3.87

**Table 3:** Stacking sequence of the CFRP/TC4 joint.

NO.	Specimen	Stacking Sequence	Stiffness Coefficient, A_11_ (N/mm)
1	BL	[45/0/−45/90/45/0_3_/−45/90/45/0_2_/−45]_s_	2.526 × 10^8^
2	CP	[(90/0)_7_]_s_	2.351 × 10^8^
3	He16	[45/−45/0/16/32/…/144/160/180]_s_	1.941 × 10^8^
4	He20	[−80/−60/…/180]_s_	1.961 × 10^8^
5	He30	[−30/0/30/…/180]_s_	2.108 × 10^8^
6	He10	[45/55/65/…/175/180]_s_	1.442 × 10^8^

**Table 4:** Main details of simulation results.

NO.	Specimen	Peak Load (kN)	Displacement at Peak Load (mm)	Energy Absorption at 6 mm (J)
1	BL	11.40	3.05	29.02
2	CP	8.12	2.22	16.41
3	He16	10.15	2.55	27.19
4	He20	9.25	3.99	23.74
5	He30	9.87	3.45	26.93
6	He10	9.31	2.78	23.65

## Data Availability

The original contributions presented in this study are included in the article. Further inquiries can be directed to the corresponding author.
